# A community effort for COVID-19 Ontology Harmonization

**Published:** 2022-01-28

**Authors:** Asiyah Yu Lin, Yuki Yamagata, William D. Duncan, Leigh C. Carmody, Tatsuya Kushida, Hiroshi Masuya, John Beverley, Biswanath Dutta, Michael DeBellis, Zoë May Pendlington, Paola Roncaglia, Yongqun He

**Affiliations:** 1 National Human Genome Research Institute, NIH, Bethesda, MD, USA; 2 RIKEN, Japan; 3 Lawrence Berkeley National Laboratory, Berkeley, CA, USA; 4 The Jackson Laboratory, Bar Harbor, ME, USA; 5 Northwest University, Evanston, Il, USA; 6 Indian Statistical Institute Bangalore Centre, India; 7 Individual Consultant and Researcher, San Francisco, CA, USA; 8 European Molecular Biology Laboratory, European Bioinformatics Institute (EMBL-EBI), Wellcome Genome Campus, Hinxton, Cambridge CB10 1SD, UK.; 9 University of Michigan Medical School, Ann Arbor, MI, USA.

**Keywords:** Knowledge integration, COVID-19, SARS-CoV-2, ontology, harmonization

## Abstract

Ontologies have emerged to become critical to support data and knowledge representation, standardization, integration, and analysis. The SARS-CoV-2 pandemic led to the rapid proliferation of COVID-19 data, as well as the development of many COVID-19 ontologies. In the interest of supporting data interoperability, we initiated a community-based effort to harmonize COVID-19 ontologies. Our effort involves the collaborative discussion among developers of seven COVID-19 related ontologies, and the merging of four ontologies. This effort demonstrates the feasibility of harmonizing these ontologies in an interoperable framework to support integrative representation and analysis of COVID-19 related data and knowledge.

## 1. Introduction

Despite the development and distribution of effective COVID-19 vaccines, COVID-19 pandemic remains a challenge to overcome. The sheer volume of data collected by researchers, the speed at which it is generated, range of its sources, quality, accuracy, and need for assessment of usefulness, results in complex, multidimensional datasets [1], often annotated in specific terminologies and coding systems by researchers in distinct disciplines. The value of cross-discipline meta-data analysis is obvious, and evident in the present pandemic. However, with the extensive COVID-19 research, we face a big challenge of *data silos*, which significantly undermine interoperability, meta-data analysis, reproducibility, pattern identification, and discovery and reusability across disciplines [2].

Ontologies - interoperable, logically well-defined, controlled vocabularies representing common entities and relations across disciplines - is a well-known solution to data silo problems. Ontologies are widely used in bioinformatics and biomedical data standardization, supporting data integration, sharing, reproducibility, and automated reasoning. To meet different needs for COVID-19 studies, different groups of ontology developers have worked separately since the start of the pandemic, resulting in the development of several COVID-19 ontologies. A lack of coordination among these groups would risk the proliferation of COVID-19 ontologies using distinct, potentially non-interoperable, vocabularies.

The Workshop on COVID-19 Ontologies (WCO-2020) held on Oct. 23 and Oct. 30, 2020 brought the developers from international groups to report their efforts on building COVID-19 related ontologies. To harmonize heterogeneous knowledge and data for better COVID-19 study, the workshop attendees formed a COVID-19 Ontology Harmonization Working Group (WG) and discussed the ways to harmonize these related ontologies. This paper reports the current results of the harmonization effort conducted by the WG.

## 2. Scope and Methods

In this study, the following seven COVID-19 related ontologies were covered in the ontology harmonization process by the COVID-19 Ontology Harmonization Working Group:

1. Virus Infectious Disease Ontology (VIDO) [3]
2. Coronavirus Infectious Disease Ontology (CIDO) [4]
3. COVID-19 Infectious Disease Ontology (IDO-COVID-19) [5]
4. Controlled Vocabulary for COVID-19 (COVoc)
5. Homeostasis imbalance process ontology (HoIP) [6]
6. Medical Action Ontology (MAxO)
7. Ontology for collection and analysis of COviD-19 data (CODO) [7]

Each of the above ontologies has their own scope and purpose. Three ontologies: Virus Infectious Disease Ontology (VIDO), Coronavirus Infectious Disease Ontology (CIDO), and COVID-19 Infectious Disease Ontology (COVID-19-IDO) all extend the Infectious Disease Ontology (IDO) [5].

The mission statement of the COVID-19 Ontology Harmonization WG is to harmonize different COVID-19 related ontologies to support COVID-19 related data and knowledge interoperability. To achieve the mission, WG members held regular virtual Zoom meetings and communicated through emails. We identified overlapping domains or subdomains from different ontology groups and built consensus on ontology terms needed to characterize specific COVID-19 related entities.

### 2.1 VIDO

VIDO (https://bioportal.bioontology.org/ontologies/VIDO) is an extension of the IDO designed to bridge IDO - which is composed of terms common to any scientific investigation of infectious disease - to virus-specific ontologies. As such, VIDO follows OBO Foundry guidelines closely. VIDO is composed of terms common to any investigation of viral infectious diseases, including virus classification, virus infection epidemiology, pathogenesis, and treatment. For example, VIDO defines terms such as virus, prion, viricide, virus infection incidence, and so on.

### 2.2 CIDO

By extending IDO and other OBO ontologies including the Ontology for Biomedical Investigations (OBI), CIDO (https://github.com/cido-ontology/cido) is developed to cover coronavirus infectious diseases including their etiology, transmission, epidemiology, host-coronavirus interaction, pathogenesis, diagnosis, prevention, and treatment. CIDO covers SARS-CoV, SARS-CoV-2, and MERS-CoV, and other coronavirus strains that cause common human cold.

### 2.3 COVID-19-IDO

COVID-19-IDO (https://bioportal.bioontology.org/ontologies/IDO-COVID-19), which was created by the developers of VIDO, is a direct extension of VIDO. As such, IDO-COVID-19 covers the epidemiology, classification, pathogenesis, and treatment of terms used to represent infection by the SARS-CoV-2 virus strain and the associated COVID-19 disease.

### 2.4 COVoc

Controlled Vocabulary for COVID-19 (COVoc) (https://github.com/EBISPOT/covoc) is an application ontology created in collaboration between the European Bioinformatics Institute (EMBL-EBI) and the Swiss Institute of Bioinformatics (SIB) in March 2020. Its primary use case is to enable seamless annotation of biomedical literature to core databases and ELIXIR tools (ELIXIR is a European-wide intergovernmental organization for life sciences). The ontology covers 9 axes related to the COVID-19 pandemic (biomedical vocabulary, cell lines, chemical entities, clinical trials, conceptual entities, diseases and syndromes, geographic locations, organisms, and proteins and genomes). COVoc utilizes existing OBO ontologies where possible to augment connections to other useful resources such as the COVID-19 Data Portal (https://www.covid19dataportal.org/).

### 2.5 CODO

Ontology for Collection and Analysis of COviD-19 Data (CODO) (https://w3id.org/codo, https://github.com/biswanathdutta/CODO) is a formal Ontology for collection and analysis of COVID-19 data [8]. The goal of the ontology was to collect data about the pandemic so that researchers could answer questions, for example about infection paths based on information about relations between patients, clusters, geography, time, comorbidities, etc. The current CODO 1.3 primarily provides the terms and relations for representing COVID-19 data and information, such as epidemiology, clinical findings, etiology, diagnosis, treatment facility, comorbidity, including the statistical data on disease spread and casualty by space and time, and resource requirements. The developed ontology can be used by the various agencies, namely doctors, hospitals, policy-makers, government agencies, application developers, etc. for various purposes, such as for developing applications, like search, question-answering systems, risk detection systems; for document annotation; for developing knowledge graph, etc. The ontology was designed by analysing disparate COVID-19 data sources such as datasets, literature, services, government published COVID-19 guidelines, WHO literature, etc.

### 2.6 HoIP

Homeostasis imbalance process ontology (HoIP) (https://bioportal.bioontology.org/ontologies/HOIP) focuses on homeostatic imbalances between virus action and innate defense processes and covers the causal relationship of organelle/cellular/organ processes from early stage to clinical manifestation in COVID-19. The design patterns between CIDO and HoIP have now been aligned after shared discussion and communication.

### 2.7 MAxO

Medical Action Ontology (MAxO), launched in the spring of 2020, is a broad ontology that provides a structured vocabulary to medical procedures, interventions, therapies, treatments, or clinical recommendations. MAxO was designed to provide a thorough resource for annotating medical actions to diseases, particularly rare diseases. Given the broad nature of MAxO and the timing of the ontology development, much of the hierarchy was added with a keen awareness of the diagnostics and treatment of SARS-CoV-2. While there are no COVID-19-specific terms, terms like ‘ventilation with proning’ (MAXO:0000619) and ‘clinical RNA detection testing’ (MAXO:0000592) were added to annotate COVID-19 clinical data sets. To capture the relationship between treatments and diseases, a new tool, Phenotypic Observation Explication Tool (POET), was developed to establish a relationship between MAxO, Human Phenotype Ontology (HPO), and Mondo Disease Ontology (Mondo) terms. This tool will allow researchers to actively participate in annotating COVID-19 data sets or other diseases in their expertise. MAxO annotations and the POET tool will be available on the HPO website (hpo.jax.org) by 2022.

## 3. Ontology Overlapping and Term Reuse

The ontology harmonization process started from identifying the scopes and development methods by different ontologies covered in this work. We found that instead of reinventing the wheel, each ontology has imported and reused many terms from other ontologies where possible (Table 1). The top 1 reused ontologies (reused in six out of the seven ontologies) are: OBI, UBERON, CL, GO Biological process,ChEBI, PRO, and RO. The top 2 reused ontologies (reused in five out of the seven ontologies) are BFO, NCBI taxon, symptom ontology and Vaccine Ontology. Many of these reused ontologies are Open Biomedical and Biological Ontologies (OBO) Foundry [8] ontologies.

## 4. Ontology Alignment and Harmonization

**Figure 1:**
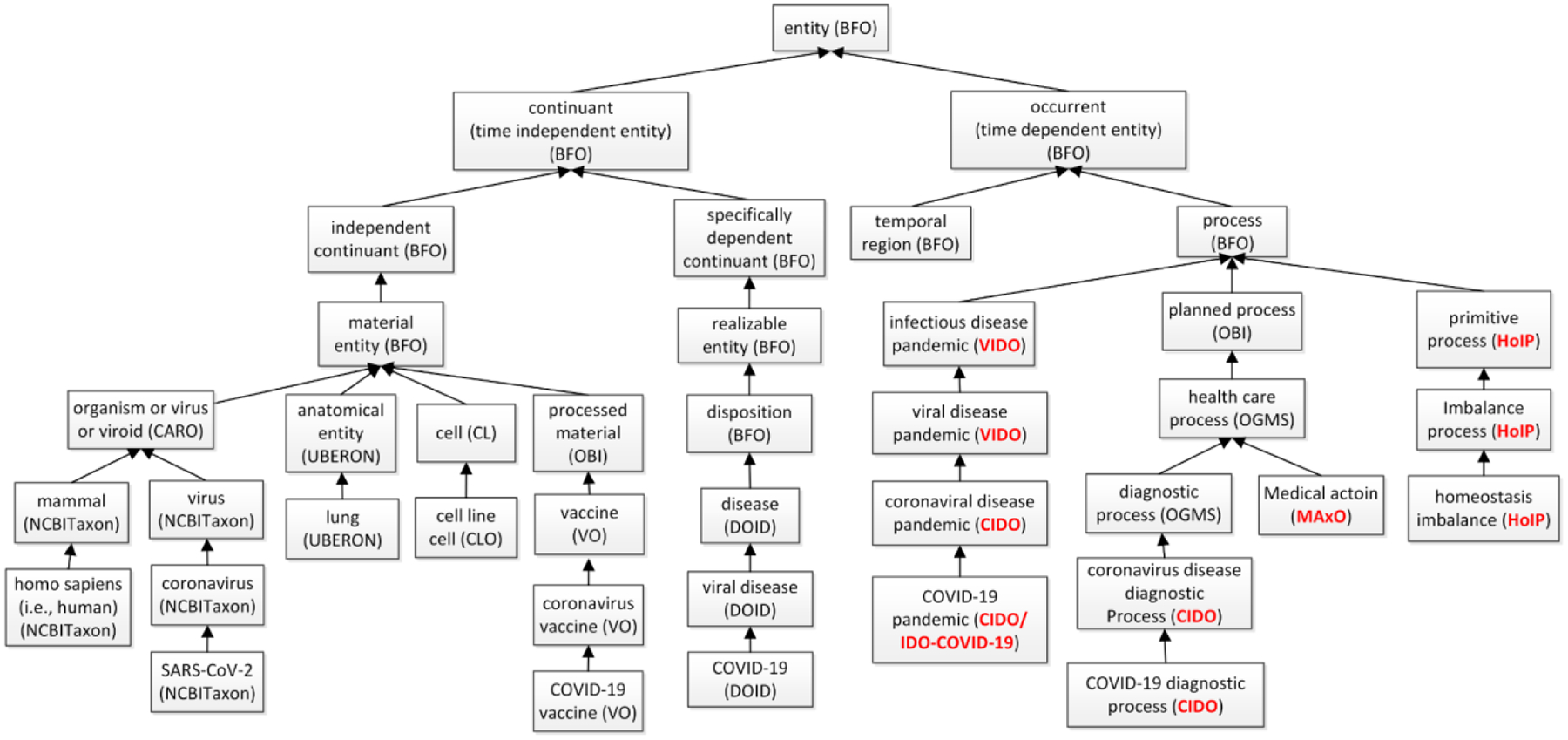
Hierarchical representation of selected terms from different ontologies that are harmonized under the BFO upper level ontology. The red colors represent ontologies focused in this ontology harmonization study. Terms from many ontologies such as BFO, NCBITaxon, and VO have been used by our ontologies as well.

Given that most of the 7 ontologies follow the OBO Foundry ontology development principles, such as reusing terms defined in OBO foundry ontologies, Our harmonization exercise found that these ontologies can be aligned under the Basic Formal Ontology (BFO) upper level ontology (Figure 1). Figure 1 below shows how VIDO, CIDO, IDO-COVID-19, MAxO and HoIP can fit into BFO’s structure.

The relationship between CIDO and IDO-COVID-19 provides an example of precisely the sort of distinct overlapping ontology development efforts our working group was created to address. Via this alignment exercise and observing the scope of CIDO appears broad enough to include IDO-COVID-19, our working group has decided to incorporate the latter ontology into CIDO. Incorporation of terms from IDO-COVID-19 into CIDO will, moreover, strengthen the logical relationship between CIDO and VIDO, given how closely related VIDO and IDO-COVID-19 are.

The HoIP developers are working on mapping and aligning with all GO process terms. Concerning harmonization, HoIP ontology has started to compare their processual entities to those in CIDO. For example, although the labels of ‘SARS-CoV-2 entry to cell’ (CIDO:0000088) and ‘viral entry into host cell [COVID-19]’ (HoIP:0037063) are different, as the HoIP entity is described using object property restriction (‘has agent’ some SARS-CoV2), it can be mapped to correspondent CIDO term. As an application ontology, the COVoc developers rely on CIDO developers to create new terms, and COVoc imports and reuses CIDO for their application purpose. At the time of writing, CODO developers started to align the current build to BFO as its upper ontology, which increases the future possibilities of better alignment.

## 5. Discussions

While ontology creates a common language and reduces the work of mapping, the emergence of multiple ontologies may form individual silos by themselves. Given the report of many COVID-19 related ontologies, our COVID-19 Ontology Harmonization WG provided a timely effort to collaboratively identify the overlapping between different ontologies and achieve the harmonization of seven ontologies. Currently, seven ontologies have very different perspectives due to their use cases. Entities within these seven ontologies are defined heterogeneously and described in various ways with various granularities. One should align not only the same URIs but also the meaning (semantics) of the entities. Therefore, it is necessary to investigate and compare entities among ontologies carefully, such as definition, superclass, logical restrictions, and related entities. Towards the formal alignment of these ontologies, we plan to clarify and make explicit the relationships such as equivalent class among the ontologies.

Members of the COVID-19 Ontology Harmonization WG made substantial efforts to characterize SARS-CoV-2 and COVID-19 data in a collaborative, computationally tractable, responsible manner. These ontologies are also being used in different use case studies, supporting productive and interoperable COVID-19 research.

The WG has also recognized many future challenges such as funding, resource and time commitment, and challenging infrastructure development. The WG members are pleased to the willingness to join the harmonization work is high, and more interested parties are joining the effort. The WG aims to continue the collaborative effort to further support the active COVID-19 research, leading to enhanced public health.

## Figures and Tables

**Table 1.** Ontology term reuse by COVID-19 related ontologies

Ontology	Domain	VIDO	CIDO	COVID-19-IDO	HoIP	CODO	MAxO	COVoc
BFO	Upper ontology	Yes	Yes	Yes	Yes	Yes		
IAO	Information content		Yes	Yes	Yes			Yes
OBI	Data item		Yes	Yes	Yes	Yes	Yes	Yes
NCBI taxon	Taxonomy	Yes	Yes	Yes	Yes			Yes
UBERON	Anatomical structure	Yes	Yes	Yes	Yes		Yes	Yes
CL	Cell	Yes	Yes	Yes	Yes		Yes	Yes
GO	Biological process	Yes	Yes	Yes	Yes		Yes	Yes
PATO	Phenotype		Yes		Yes		Yes	
HPO	Phenotype		Yes		Yes		Yes	Yes
ChEBI	Chemical compound	Yes	Yes	Yes	Yes		Yes	Yes
PRO	Protein	Yes	Yes	Yes	Yes		Yes	Yes
HGNC	Gene		Yes					
OGG	Gene				Yes			
DO	Disease		Yes		Yes	Yes		
MONDO	Disease						Yes	Yes
SNOMED CT	Disease					Yes		
NDF-RT	Disease/Finding		Yes					
Symptom	Symptom	Yes	Yes	Yes	Yes	Yes		
Vaccine Ontology	Vaccine	Yes	Yes	Yes	Yes	Yes		
RO	Relational ontology	Yes	Yes	Yes	Yes		Yes	Yes
